# β-Cyclodextrin-Grafted Polypyrrole–Rhodamine B Nanoplatforms for Drug Delivery and Image-Guided Photothermal Therapy In Vitro

**DOI:** 10.3390/ma18235313

**Published:** 2025-11-25

**Authors:** Shasha Hong, Yuan Jiao, Ruyu Li, Peng Lei, Chuan Dong, Shang Guo, Shaomin Shuang

**Affiliations:** 1 Shanxi Institute for Functional Food, Shanxi Agricultural University, Taiyuan 030031, China; sshong@sxau.edu.cn; 2 College of Environment and Ecology, Taiyuan University of Technology, Jinzhong 030600, China; 3 Institute of Environmental Science, College of Chemistry and Chemical Engineering, Shanxi University, Taiyuan 030006, China

**Keywords:** β-cyclodextrins, polypyrrole, rhodamine B, drug delivery, thermal sensing

## Abstract

Synergetic therapeutic study using multifunctional nanoplatforms has been developed as an innovative modality for effective cancer treatment to improve the clinical efficiency of anticancer drugs and reduce severe off-target side effects. Herein, an artificial nanoplatform (denoted as PPy-RhB-PDA-CD-LA) was prepared by grafting β-cyclodextrin (β-CD) derivatives and lactobionic acid (LA) on the surface of rhodamine B (RhB)-doped polypyrrole nanoparticles (PPy-RhB NPs) using polydopamine (PDA) as the intermediate linker. Doxorubicin (DOX) was selected and successfully loaded onto the nanoplatforms with a high loading content of 327 mg/g. Furthermore, significant NIR light-triggered release of DOX was observed in a weak acidic tumor microenvironment. The nanoplatform exhibited superior photostability with a high photothermal effect of 51.7% under irradiation by a 808 nm laser and a competent temperature sensitivity (SR is 1.44% °C^−1^) under a single wavelength excitation. MTT assay against SMMC-7721 cells clearly illustrated that the nanoplatform had low cytotoxicity at a high level (200 μg/mL) after 24 h and high therapeutic efficacy of chemo-phototherapy. Thus, it is highly promising for use in biomedical applications.

## 1. Introduction

Despite decades of extensive research and substantial efforts, cancer treatment continues to face significant challenges due to its inherent biological complexities and unpredictable clinical behavior [1]. Chemotherapy remains one of the effective anticancer strategies, and widely used agents, such as doxorubicin (DOX), camptothecin (CPT), and paclitaxel (PTX), play a crucial role in therapeutic regimens [2,3]. However, the clinical utility of these chemotherapeutic drugs is often hampered by limitations, such as poor bioavailability, systemic toxicity, and the emergence of drug resistance. Thus, multifunctional nanoplatforms have been developed to address these issues, aiming to optimize drug delivery and enhance anticancer efficacy [4,5]. Currently, β-cyclodextrin (β-CD) and derivative-based multifunctional nanoplatforms have attracted lots of attention [6,7]. β-CD is a macrocyclic oligosaccharide composed of α-(1-4) linked glucose units, with a hydrophilic outer surface and a hydrophobic inner cavity. This allows it to encapsulate hydrophobic chemotherapeutic drugs through non-covalent interactions. Through in-depth research on β-CD, researchers have discovered that integrating β-CD with active targeting ligands and biocompatible polymers into multifunctional nanoplatforms can significantly improve its properties, including targeted drug delivery, high drug-loading capacity, and stimuli-responsive drug release [8,9]. Lactobionic acid (LA), a widely recognized targeting ligand, exhibits specific affinity for asialoglycoprotein receptors (ASGPR), which are selectively overexpressed on hepatocytes [10]. Previous studies have demonstrated that LA-based multifunctional nanoplatforms can achieve effective hepatocellular carcinoma targeting and promising tumor inhibition [11,12]. Accordingly, the conjugation of LA with β-CD derivatives is designed to potentially enhance liver-specific drug delivery through ASGPR-mediated pathways.

Additionally, polymeric materials have also been widely used for β-CD modification to regulate drug release under different disease/physiological conditions [13]. Our previous work revealed that the developed β-CD-based nanoplatform integrated with polypyrrole (PPy), an near-infrared (NIR) light-absorbing photothermal conductive polymer, enabled effective pH and NIR light-controlled release of DOX [14]. By applying this strategy, the constructed nanoplatform is expected to achieve a synergistic combination of chemotherapy with photothermal therapy (PPT) effects [15]. Since Goldman pioneered laser-based tumor removal in 1966 [16], PTT has gained widespread interest in medicine owing to its intrinsic advantages: minimally invasive, low toxicity, simple operation, and rapid recovery [17]. For effective PTT, the temperature of the target lesion must be carefully regulated to avoid overheating, which could lead to irreversible harm to adjacent normal tissues. Consequently, the integration of temperature-sensing functionality into PPT to realize in situ and real-time temperature measurement at microscopic levels is indispensable [18,19,20].

**Figure 1:**
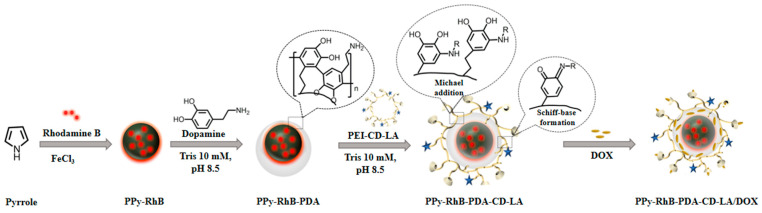
Schematic illustration of the synthesis of PPy-RhB-PDA-CD-LA.

Inspired by these investigations, a multifunctional nanoplatform was designed to combine controlled drug release, photothermal conversion, and temperature sensing through layer-by-layer assembly. As shown in Figure 1, firstly, rhodamine B (RhB), as the temperature-sensing probe [21], was doped into the PPy nanoparticles (referred to as PPy-RhB) using a chemical oxidative polymerization method. Secondly, polydopamine (PDA), an FDA-approved polymer renowned for its good biocompatibility and biodegradability [22], was used as the surface modifier for coating on the surface of PPy-RhB to enhance its photothermal performance and drug-loading capacity (referred to as PPy-RhB-PDA). Thirdly, polyethylenimine (PEI) was functionalized with β-CD and LA to generate PEI-CD-LA, designed to potentially enhance drug delivery efficiency while utilizing β-CD for drug encapsulation. Lastly, PEI-CD-LA was further modified on the surface of PPy-RhB-PDA to develop the final multifunctional nanoplatform (referred to as PPy-RhB-PDA-CD-LA) through Michael addition and Schiff base reactions. Following the characterization of physicochemical properties, evaluation of photothermal performance, in vitro release kinetics, and temperature-sensing capabilities, the thermal fluorescence-sensing and synergistic chemo-phototherapy effects of the engineered nanoplatform were further investigated using cellular models.

## 2. Materials and Methods

### 2.1. Materials

N-Hydroxysuccinimide (NHS), N-(3-dimethylaminopropyl)-N′-ethylcarbodiimide (EDC), pyrrole, DOX, dopamine (DA), and tris hydrochloride (Tris-HCl) were purchased from Shanghai Aladdin Biochemical Technology Co., Ltd. (Shanghai, China). Iron (III) chloride hexahydrate (FeCl_3_·6H_2_O) was purchased from Tianjin Chemicals Factory (Tianjin, China). Polyvinylpyrrolidone (PVP) was purchased from Beijing Chemicals Factory (Beijing, China). RhB was purchased from TCI (Shanghai) Development Co., Ltd. (Shanghai, China). LA was purchased from Shanghai Macklin Biochemical Technology Co., Ltd. (Shanghai, China). All chemicals were commercially purchased and used without further purification.

### 2.2. Characterization

^1^H NMR spectrum was obtained using a Bruker Avance III 600 MHz nuclear magnetic resonance spectrometer (Bruker, Bremen, Germany). High-resolution transmission electron microscope (TEM) images were obtained using a Tecnai G2 F20 TEM (FEI, Hillsboro, OR, USA). Fourier transform infrared (FT-IR) spectra were recorded on a Tensor-27 (Bruker, Germany) spectrometer using KBr pellets. UV-Vis absorption spectra were measured with a U-2910 spectrophotometer (Hitachi, Tokyo, Japan). The fluorescence intensity was collected using an F-4500 fluorescence spectrophotometer (Hitachi, Japan). The hydrodynamic size, polydispersity, and zeta potential were further measured through dynamic light scattering (DLS) using a Malvern Nano ZS90 system (Malvern Panalytical, Malvern, UK). UV-Vis-NIR absorbance spectra were measured with a Lambda950 spectrophotometer (PerkinElmer, Shelton, CT, USA).

### 2.3. Preparation of PPy-RhB-PDA-CD-LA

Synthesis of PPy-RhB. The synthesis of PPy-RhB was carried out via an oxidative polymerization method in the presence of RhB, based on a previous report [23]. Briefly, 0.4 g of PVP and 0.3 g of FeCl_3_·6H_2_O were mixed, and then 50 mL of deionized water was added. The temperature of the mixture was maintained at 0~5 °C using an ice bath. After stirring for 0.5 h, 0.07 mL of pyrrole monomer was added into the mixture drop by drop. Finally, the mixture was polymerized in the ice bath for 4 h to obtain PPy-RhB with a dark purple color. The resulting PPy-RhB was collected through centrifugation and washed with deionized water and then redispersed in deionized water for further experiments.

Synthesis of PPy-RhB-PDA. The PDA thin layer was modified on the surface of PPy-RhB NPs by adjusting the amount of dopamine monomer under weak alkaline conditions. In brief, PPy-RhB and dopamine (mass ratios of 50:1, 25:1, 10:1, and 5:1) were mixed into 20 mL of Tris-HCl buffer (10 mM, pH 8.5). After stirring for 12 h at room temperature, the mixture was centrifuged and washed three times with deionized water. Then, the obtained PPy-RhB-PDA was redispersed in deionized water for later use.

Synthesis of PPy-RhB-PDA-CD-LA. PEI-CD-LA as the outermost layer was modified on the surface of PPy-RhB-PDA through Michael addition and Schiff base reactions [24]. The detailed synthesis process and characterization data of PEI-CD-LA are provided in the Supporting Information. Then, 25 mg of PPy-RhB-PDA was dispersed in 50 mL of Tris-HCl buffer (10 mM, pH 8.5). Next, 50 mg of PEI-CD-LA was added and continuously stirred for 24 h at room temperature. The product was collected through centrifugation, washed with deionized water, and then redispersed in deionized water for further use.

### 2.4. In Vitro Photothermal Efficiency and Temperature Sensing

To assess the photothermal effect of PPy-RhB-PDA-CD-LA, PPy-RhB-PDA-CD-LA (0.8 mg/mL) was irradiated by an 808 nm near-infrared laser (MDL-Ⅲ-808nm-2.5W, Changchun New Industries Optoelectronics Technology Co., Ltd., Changchun, China) with various output energies for 10 min, and an equivalent amount of deionized water was set as the control group. The output power was remeasured using a Thorlabs PM100D power meter equipped with both Thorlabs S425C-L and S130C probes (Thorlabs Inc., Newton, NJ, USA). In addition, PPy-RhB-PDA-CD-LA (0.8 mg/mL) was continuously irradiated by the 808 nm laser (1.226 W) for 10 min, followed by natural cooling to room temperature. The photothermal stability of PPy-RhB-PDA-CD-LA was tested by repeatedly turning on and off the laser for five cycles. Meanwhile, the temperature profile was monitored in 20 s intervals with a thermal couple probe during the whole procedure.

For in vitro temperature sensing, PPy-RhB-PDA-CD-LA (0.8 mg/mL) was heated, and the fluorescence intensity was measured using a fluorescence spectrometer. The fluorescence spectra of PPy-RhB-PDA-CD-LA were recorded from 565 to 700 nm under excitation at 545 nm. The excitation and emission slits were both 5 nm. The temperature changes were recorded in real time with a thermal couple probe.

### 2.5. Drug Loading and Release

As a commonly used anticancer drug with limited bioavailability, DOX was chosen as the model drug to evaluate the drug loading capacity of PPy-RhB-PDA-CD-LA. First, 4 mg of PPy-RhB-PDA-CD-LA was dissolved in 20 mL of phosphate-buffered saline (0.01 M PBS, 7.4) containing DOX with different concentrations. After being shaken at 37 °C in the dark for 24 h, PPy-RhB-PDA-CD-LA/DOX was collected through centrifugation and washed with PBS. Then, the supernatant was spectrophotometrically measured at 480 nm to determine the amount of DOX. The drug loading content was calculated as follows:

$$q_{e}=\frac{(C_{0}-C)V}{m}$$

where *C*_0_ and *C* stand for the initial and final concentrations of DOX, *V* for the volume of DOX solution, and *m* for the mass of PPy-RhB-PDA-CD-LA.

The release of DOX from PPy-RhB-PDA-CD-LA/DOX was assessed using a dialysis method as follows. First, 5 mg of samples was dispersed in different pH levels of PBS (0.01 M, 5.0 and 7.4), followed by transfer in dialysis bags with a molecular weight cutoff of 3500 Da. The bags were separately soaked in 50 mL of PBS with the corresponding pH values and kept in the air bath thermostatic shaker with shaking at 150 rpm and 37 °C. At predetermined time points, 2 mL of the dialysate was taken, and an equal volume of fresh PBS was added immediately. The absorbance of dialysate was determined at 480 nm using an UV-vis spectrophotometer. Meanwhile, to further investigate the effect of NIR laser irradiation on the release of DOX, the same concentration of the PPy-RhB-PDA-CD-LA/DOX suspension was continuously irradiated with the 808 nm NIR laser (1.226 W) for 15 min, followed by removing 2 mL of dialysate from the sample, while all other conditions remained unchanged.

### 2.6. In Vitro Cytotoxicity Assay

MTT assays were conducted using the human hepatocellular carcinoma cell line SMMC-7721 (purchased from the Type Culture Collection of the Chinese Academy of Sciences, Shanghai, China) to evaluate the cytocompatibility of PPy-RhB-PDA-CD-LA, free DOX, and PPy-RhB-PDA-CD-LA/DOX [25]. The cells were seeded into a 96-well plate, cultured overnight, and then treated with different concentrations of PPy-RhB-PDA-CD-LA (25, 50, 100, 150, and 200 μg/mL), DOX (0.01, 0.1, 1, 5, and 10 μg/mL), and PPy-RhB-PDA-CD-LA/DOX (DOX concentrations of 0.01, 0.1, 1, 5, and 10 μg/mL) for 24 h. Furthermore, to qualitatively evaluate the photothermal therapeutic efficiency of PPy-RhB-PDA-CD-LA and PPy-RhB-PDA-CD-LA/DOX in vitro, SMMC-7721 cells were cultured and treated with different concentrations of PPy-RhB-PDA-CD-LA and PPy-RhB-PDA-CD-LA/DOX for 12 h. After irradiation with an 808 nm laser (1.226 W) for 2 min, the cells were further incubated for 12 h. Six replicates were included for each treatment group. Finally, MTT assay was performed after further incubation of cells with MTT solution for 4 h. Cell viability was evaluated by measuring the absorbance of the suspension at 570 nm using an Infinite 200 PRO enzyme-linked immunosorbent assay reader (Tecan Trading, AG, Männedorf, Switzerland).

### 2.7. Cellular Imaging Analysis

To assess the cellular uptake of free DOX, PPy-RhB-PDA-CD-LA/DOX, and PPy-RhB-PDA-CD-LA by SMMC-7721 cells, the cells were seeded in the confocal dish and incubated with free DOX (0.1 μg/mL), PPy-RhB-PDA-CD-LA/DOX (DOX concentration of 0.1 μg/mL), and PPy-RhB-PDA-CD-LA (100 μg/mL) for 30 min and 3 h. Subsequently, the cell culture medium was removed, the cells were washed three times with cold PBS, and then they were stained with Hoechst 33258 for 10 min in the dark to label the nuclei. The fluorescence images were observed through confocal laser scanning microscopy (CLSM; Zeiss LSM880, Jena, Germany).

The fluorescence changes of PPy-RhB-PDA-CD-LA during photothermal therapy in vitro were investigated. SMMC-7721 cells were incubated with 100 μg/mL of PPy-RhB-PDA-CD-LA for 12 h. After repeated washing with PBS, the confocal dish was irradiated with an 808 nm laser (1.226 W) for 0, 2, 4, 6, 8, and 10 min, and the fluorescence changes during the period were observed and analyzed through CLSM.

## 3. Results and Discussion

### 3.1. Characterization of PPy-RhB-PDA-CD-LA

**Figure 2:**
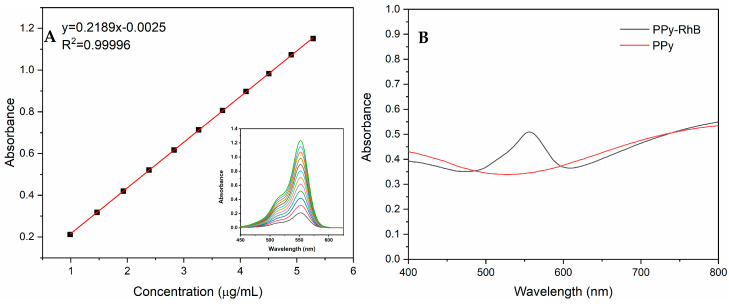
(**A**) Standard curve of RhB. (**B**) UV-vis absorption spectra of PPy and PPy-RhB.

PPy-RhB was synthesized via in situ oxidation polymerization in the presence of RhB at room temperature. As displayed in Figure 2A, the absorption peak intensity exhibited a satisfactory linear relationship to the concentration of RhB. A linear equation y = 0.2189x − 0.0025 was obtained, where y represents the absorption peak intensity of RhB and x represents the concentration of RhB. The loading capacity of PPy-RhB for RhB was determined by monitoring the absorbance of PPy-RhB (0.1951 mg/mL) and PPy (Figure 2B). Correspondingly, y = 0.505 − 0.344 = 0.161, and the concentration of RhB was calculated to be 0.7469 µg/mL based on the standard curve of RhB. The loading capacity of PPy-RhB for RhB was further calculated to be approximately 0.38% (0.7469 × 10^−3^/0.1951 ≈ 0.38%) [23].

**Figure 3:**
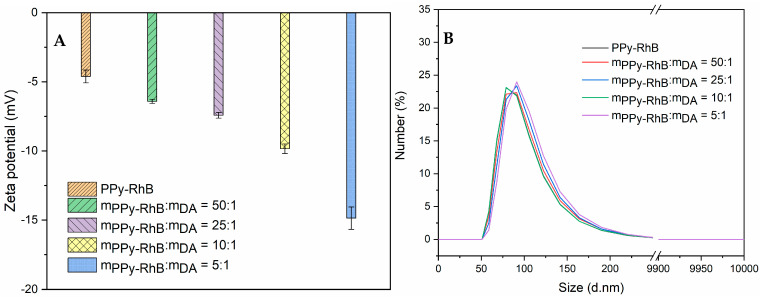
(**A**) Zeta potential of PPy-RhB-PDA. (**B**) Hydrodynamic size of PPy-RhB-PDA measured through dynamic light scattering (DLS) analysis.

The thin layer of PDA was coated on the surfaces of PPy-RhB through the oxidative self-polymerization of dopamine under alkaline conditions [26]. To further investigate the effect of the amount of PPy-RhB on the formation of PPy-RhB-PDA, the mass ratio of PPy-RhB to DA was adjusted to 1:0, 50:1, 25:1, 10:1, and 5:1. After the modification of PDA, the major components on the surface of PPy-RhB-PDA were catechol groups with deprotonated hydroxyl groups [27]. As shown in Figure 3A, a decreasing amount of PPy-RhB caused an decrease in the zeta potential of PPy-RhB-PDA. The correlative zeta potential values of PPy-RhB-PDA were −4.6 ± 0.5 mV, −6.4 ± 0.2 mV, −7.4 ± 0.2 mV, −9.8 ± 0.6 mV, and −14.9 ± 0.8 mV. Therefore, it may be suggested that more dopamine could be polymerized and deposited on the surface of PPy-RhB by simply decreasing the mass ratio of PPy-RhB to DA.

In addition, the introduction of PDA resulted in a negligible hydrodynamic diameter of PPy-RhB, as illustrated in Figure 3B and Table 1. Wu et al. [28] systematically investigated the influence of various reaction conditions (DA:NaOH molar ratio, DA concentration, reaction time, temperature, and oxidant types) on the formation pathways of PDA. They concluded that the DA:NaOH molar ratio was the most effective parameter for controlling the diameter and size distribution of PDA spheres owing to its decisive role during the initial stage of the reaction pathway. In a related study, Ball et al. [29] proposed that the DA concentration significantly affected the thickness and morphology of the deposited layer by modulating the polymerization mechanism. Our results indicated that the final diameter of the PPy-RhB-PDA was largely unaffected by the reduction in the amount of PPy-RhB, as the DA:OH^-^ ratio and the dopamine concentration were maintained at fixed values throughout the experiment. The particle size distribution within a given sample was commonly characterized by the polydispersity index (PDI). As shown in Table 1, the PDI values in solutions with varying PPy-RhB:DA mass ratios were all below 0.3, suggesting a predominantly monodisperse particle population [30].

**Figure 4:**
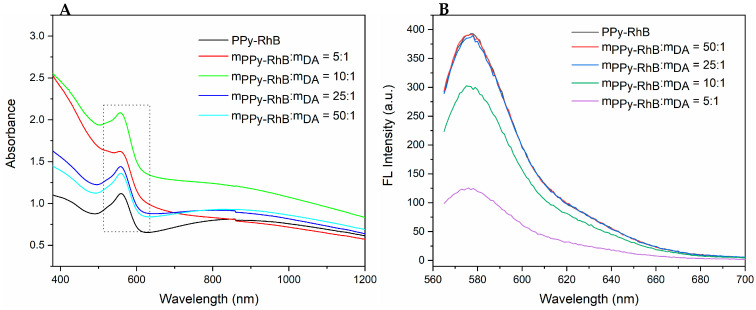
(**A**) UV-vis-NIR absorbance spectra of PPy-RhB and PPy-RhB-PDA (The dashed box indicates the typical absorbance peak of RhB). (**B**) Fluorescence spectra of PPy-RhB and PPy-RhB-PDA (λex: 545 nm).

UV-vis-NIR and fluorescence spectroscopy were used to further analyze the effects of PPy-RhB dosage on the optical properties of PPy-RhB-PDA. In the UV-vis-NIR spectra (Figure 4A), a broad band from visible to NIR region (600~1200 nm) was observed, attributed to the characteristic band of the bipolaronic metallic state of PPy [31]. The absorption spectra of PPy-RhB and PPy-RhB-PDA showed the typical absorbance peak of RhB at 545 nm, which was still exhibited after dopamine modification, indicating the successful incorporation of PPy-RhB-PDA. As illustrated in Figure 4B, PPy-RhB and PPy-RhB-PDA (m_PPy-RhB_:m_DA_ = 50:1, m_PPy-RhB_:m_DA_ = 25:1) emitted strong fluorescence at an excitation wavelength of 580 nm when the emission wavelength was 545 nm. The fluorescence intensity of PPy-RhB-PDA (m_PPy-RhB_:m_DA_ = 10:1, m_PPy-RhB_:m_DA_ = 5:1) decreased as the amount of dopamine increased within a certain range. In this work, the thin layer of PDA was mainly used to couple with the amino groups of PEI-CD-LA via Schiff-based and Michael addition reactions. Considering the effect of dopamine dosage on the fluorescence intensity of PPy-RhB-PDA and the function of PDA, a mass ratio of 25:1 (m_PPy-RhB_:m_DA_) was selected in the follow-up experiment.

**Figure 5:**
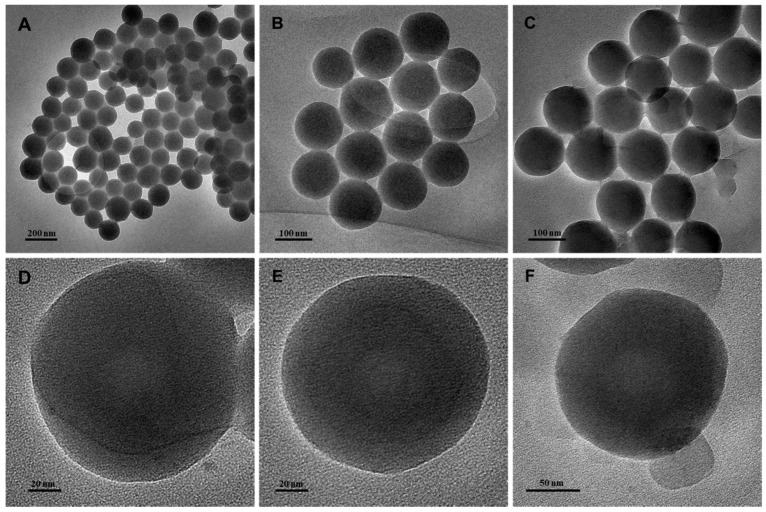
TEM images of PPy-RhB (**A**,**D**), PPy-RhB-PDA (**B**,**E**), and PPy-RhB-PDA-CD-LA (**C**,**F**).

TEM evaluation of the morphology of the synthesized PPy-RhB, PPy-RhB-PDA, and PPy-RhB-PDA-CD-LA, which displayed a regular spherical structure with good dispersion (Figure 5). The sizes of PPy-RhB, PPy-RhB-PDA, and PPy-RhB-PDA-CD-LA were analyzed using Image J1 software as 127.1 nm, 129.4 nm and 133.0 nm, respectively. The corresponding size distribution is provided in Figure S4. After layer-by-layer accretion, the morphology of the synthesized nanoparticles showed negligible changes, and the size increased by approximately 2.3 nm and 3.6 nm, sequentially.

**Figure 6:**
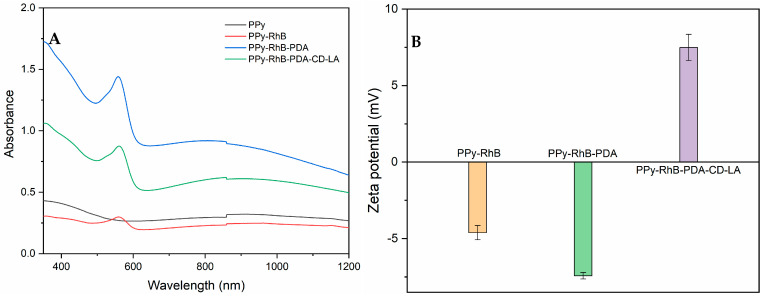
(**A**) UV-vis-NIR absorbance spectra of PPy, PPy-RhB, PPy-RhB-PDA, and PPy-RhB-PDA-CD-LA. (**B**) Zeta potential of PPy-RhB, PPy-RhB-PDA, and PPy-RhB-PDA-CD-LA.

Figure 6A shows the UV-vis-NIR spectra of PPy, PPy-RhB, PPy-RhB-PDA, and PPy-RhB-PDA-CD-LA, which revealed that the prepared PPy-RhB-PDA-CD-LA exhibited a broad band from 600 nm to 1200 nm with strong absorption (identical to the absorption spectra of PPy-RhB and PPy-RhB-PDA). In addition, the typical absorbance peak of RhB at 545 nm was still shown in the UV-vis-NIR spectra of PPy-RhB-PDA-CD-LA. Figure 6B showed that the zeta potential values of PPy-RhB, PPy-RhB-PDA, and PPy-RhB-PDA-CD-LA were −4.6 ± 0.5 mV, −7.4 ± 0.2 mV, and 7.5 ± 0.9 mV, respectively. The positive zeta potential of PPy-RhB-PDA-CD-LA can be attributed to the protonation of amino groups in the unreacted branches of PEI-CD-LA [32]. This finding is further supported by the structural characterization of PEI-CD-LA (Figures S2 and S3), thus confirming the successful synthesis of PPy-RhB-PDA-CD-LA.

### 3.2. Photothermal Effects of PPy-RhB-PDA-CD-LA

**Figure 7:**
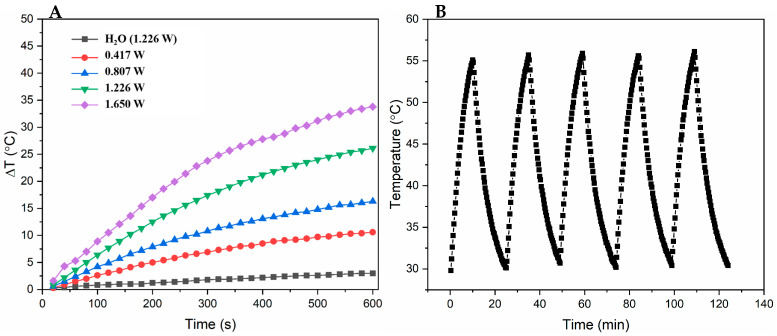
(**A**) The temperature change (ΔT) of PPy-RhB-PDA-CD-LA (0.8 mg/mL) and water with 808 nm laser irradiation at different powers for different times. (**B**) Temperature curves of PPy-RhB-PDA-CD-LA during five laser on/off cycles under irradiation using an 808 nm laser.

The photothermal properties of PPy-RhB-PDA-CD-LA were assessed under 808 nm laser irradiation. As shown in Figure 7A, 0.8 mg/mL of PPy-RhB-PDA-CD-LA solution (1.5 mL) was irradiated using an 808 nm laser at various powers (0.417, 0.807, 1.226, and 1.65 W) for 10 min, and then the temperature change (ΔT) of the solution reached 10.6, 16.3, 26.1, and 33.8 °C, respectively. In contrast, there was a minimal increase in temperature (3 °C) for deionized water irradiated at 1.226 W for 10 min. To further examine the photothermal stability of PPy-RhB-PDA-CD-LA, five cycles of NIR laser on/off were performed. A similar pattern of temperature change was observed, shown in Figure 7B, during the five sequential heating–cooling cycles. The photothermal conversion efficacy (η) of PPy-RhB-PDA-CD-LA was calculated to be 51.7% based on one of the cycles [33]. Detailed calculations are provided in the Supporting Information (Figure S6). The results show that PPy-RhB-PDA-CD-LA has superior photothermal stability and photothermal conversion ability and broad application prospects in the photothermal therapy of tumors.

### 3.3. Temperature-Dependent Fluorescence of PPy-RhB-PDA-CD-LA

**Figure 8:**
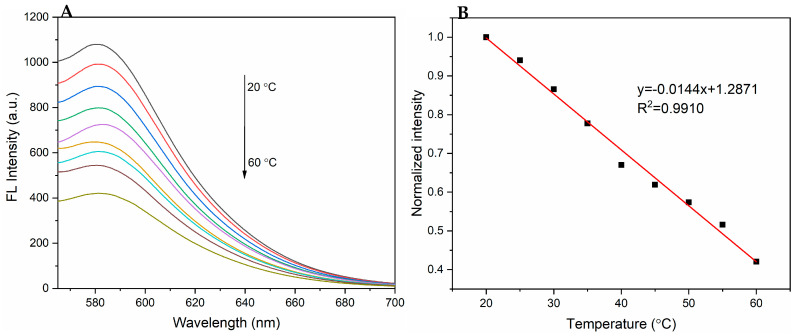
(**A**) Emission spectra of PPy-RhB-PDA-CD-LA at different temperatures (λex: 545 nm). (**B**) Calibration curve of emission intensity versus different temperatures.

The thermoresponsive properties of PPy-RhB-PDA-CD-LA were studied to explore potential applications in intracellular temperature sensing. The fluorescence spectra of PPy-RhB-PDA-CD-LA solution (1.5 mL, 0.8 mg/mL) at temperatures ranging from 20 to 60 °C are shown in Figure 8A. With the increasing temperature, the fluorescence intensity of PPy-RhB-PDA-CD-LA at 580 nm was found to decrease. A linear relationship between the fluorescence intensity and temperature was described as Ratio = −0.0144T + 1.2871 (R^2^ = 0.9910) in the range of 20–60 °C. Drawing on the literature, thermal sensitivity ($S_{R}$) was calculated using the following equation [34,35]:

$$S_{R}=\frac{d_{\mathrm{Ratio}}}{d_{T}}\frac{1}{\mathrm{Ratio}}$$

where *d_Ratio_*/*d_T_* is the slope in the temperature calibration curve of Figure 8B and $S_{R}$ is calculated to be 1.44% °C^−1^ at 20 °C. This parameter serves as an indicator of fluorescence intensity variation with temperature and exceeds the minimum signal variation required for temperature discrimination (0.5% °C^−1^) [36,37]. The PPy-RhB-PDA-CD-LA exhibited good sensing performance compared to various nanothermometers in Table S1 and provided a basis for the realization of temperature sensing during photothermal therapy.

### 3.4. Drug Loading and Releasing Properties of PPy-RhB-PDA-CD-LA

**Figure 9:**
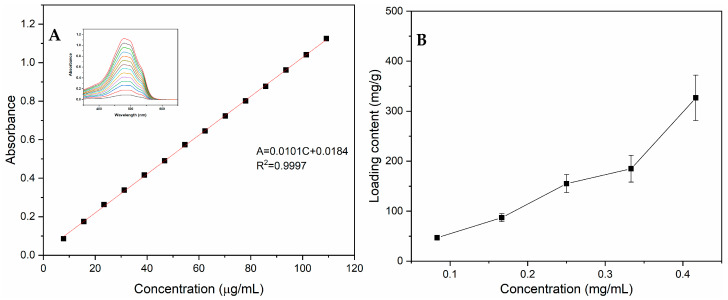
(**A**) The standard curve of DOX solutions detected at 480 nm. (**B**) Adsorption isotherm of PPy-RhB-PDA-CD-LA in the drug-loading process.

Drug loading and its release behavior stand out as essential characteristics of an effective drug carrier. The amount of DOX loaded was determined by calculating the amount of DOX in the supernatant after drug loading. A pre-established calibration curve for DOX (A = 0.0101C + 0.0184, R^2^ = 0.9997; Figure 9A) over a concentration range of 7–110 μg/mL was used (Figure 9A). As shown in Figure 9B, the drug loading content of PPy-RhB-PDA-CD-LA gradually increased with the increase in DOX concentration. At the concentration of 0.42 mg/mL, the loading capacity of PPy-RhB-PDA-CD-LA reached as high as 326.6 mg/g. An earlier study demonstrated that the anthracycline drug DOX effectively occupied the cavity of β-CD through the formation of an inclusion complex stabilized in the quinol conformation [38]. Additionally, the abundant aromatic rings on the PDA surface conferred PPy-RhB-PDA-CD-LA with exceptional drug-loading capacity for DOX through hydrogen bonding and π-π stacking interactions with its π-electron conjugated structure [39,40].

**Figure 10:**
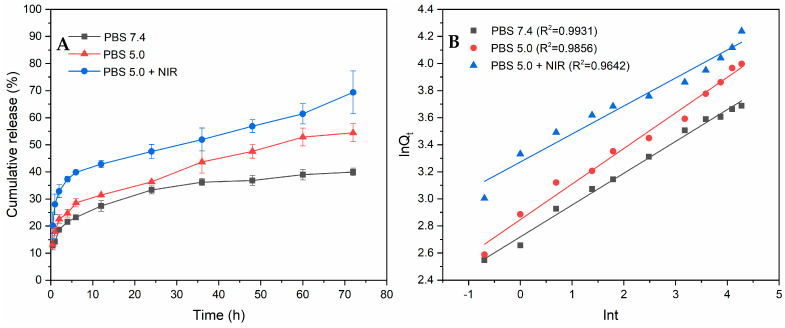
(**A**) Accumulated releasing profiles of DOX in PBS at various pH values with and without the irradiation of the 808 nm laser. (**B**) The fitting curves of accumulated releasing DOX through the Ritger–Peppas kinetic equation.

The release curves of PPy-RhB-PDA-CD-LA/DOX are illustrated in Figure 10A. Within the first 1 h, the released DOX from the PPy-RhB-PDA-CD-LA/DOX was 14.25% and 17.92% in neutral solution (pH 7.4) and acidic solution (pH 5.0), respectively. At the same time, the DOX release rate increased significantly to 28.01% in the presence of NIR irradiation. Undoubtedly, an initial burst release behavior of DOX was observed, attributable to the desorption of DOX adsorbed on the surface of the drug carrier. After immersion for 24 h and 72 h, the release rate of DOX gradually decreased, and approximately 33.34% and 39.42% of DOX fled from PPy-RhB-PDA-CD-LA/DOX under the condition of pH 7.4, respectively. In contrast, within 24 h and 72 h, the percentage of released DOX in the acidic solution was 36.31% and 54.46%, respectively. Furthermore, DOX release was triggered by laser irradiation, resulting in cumulative DOX release of 47.54% at 24 h and 69.37% at 72 h, respectively. These findings indicated that both acidity and NIR irradiation induced the release of DOX. The pH response of PPy-RhB-PDA-CD-LA/DOX could be explained by the weak association between DOX and β-CD in an acidic environment [38]. Another possible reason is the existence of positive–positive electrostatic repulsion between the DOX and the outer layer of PPy-RhB-PDA-CD-LA, as both the amino groups of PEI and the amide groups of PDA are easily protonated under acidic conditions [41]. Furthermore, the NIR irradiation response could be attributed to that the photothermal energy from the PPy-RhB-PDA-CD-LA as the NIR laser increased the thermal movement of DOX molecules and simultaneously destroyed the intermolecular forces [40].

The obtained release results were fitted using zero-order, first-order, Higuchi, and Ritger–Peppas kinetic models, as presented in Figure 10B and Table S2. Compared with other release kinetic models, the Riter–Peppas model exhibited the highest correlation coefficient (*R*^2^) value, indicating that the Riter–Peppas model was the best-fitted model. The resulting release exponent (*n*) values of the Ritger–Peppas model were 0.2352 (pH 7.4), 0.2629 (pH 5.0), and 0.2068 (pH 5.0 + NIR), respectively, and less than 0.43, implying that the release of the DOX from PPy-RhB-PDA-CD-LA/DOX conformed to Fickian diffusion mechanisms [42,43].

### 3.5. Cytotoxicity and Cell Photothermal Effect

**Figure 11:**
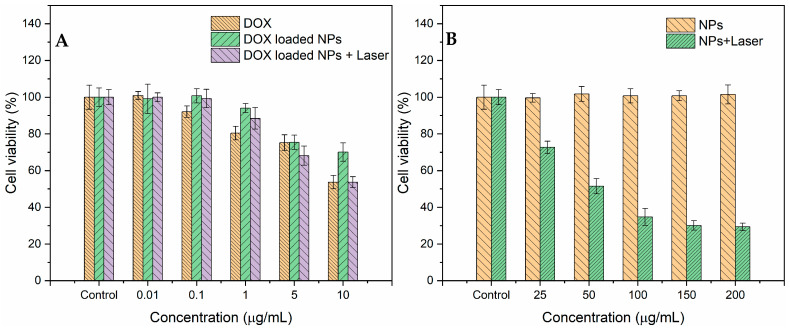
Cell viability of SMMC-7721 cells after incubation for 24 h with increased doses of DOX, PPy-RhB-PDA-CD-LA/DOX (**A**), and PPy-RhB-PDA-CD-LA (**B**) with or without 808 nm irradiation.

The in vitro therapeutic effects of DOX and PPy-RhB-PDA-CD-LA/DOX with or without NIR light irradiation were evaluated using the MTT assay. As shown in Figure 11A, both DOX and PPy-RhB-PDA-CD-LA/DOX exhibited dose-dependent toxicity of SMMC-7721 cells in the DOX concentration range of 0.01–10 μg/mL. At the highest concentration (DOX concentration of 10 μg/mL), cell viability after DOX treatment was 53.7%, which was significantly lower than that under PPy-RhB-PDA-CD-LA/DOX treatment (70.1%), indicating that cell killing against SMMC-7721 cells was lower for PPy-RhB-PDA-CD-LA/DOX than for DOX. This characteristic could be explained by the fact that more time was needed for PPy-RhB-PDA-CD-LA cellular uptake than free DOX and the slow release of DOX [44]. In comparison, when SMMC-7721 cells were treated with PPy-RhB-PDA-CD-LA/DOX and irradiated with an 808 nm NIR laser (1.226 W) for 2 min, cell viability decreased significantly to 53.7% at a DOX concentration of 10 μg/mL. This enhanced cytotoxic effect can be attributed to the photothermal properties of PPy-RhB-PDA-CD-LA under NIR irradiation, combined with NIR light-triggered DOX release, consistent with the results presented in Figure 10A. In addition, the viability of SMMC-7721 cells was evaluated after 24 h of treatment with varying concentrations of PPy-RhB-PDA-CD-LA. The results indicated no detectable cytotoxicity even at a high concentration of 200 μg/mL (Figure 11B). In contrast, cell viability decreased significantly to 29.4% when treated with 200 μg/mL PPy-RhB-PDA-CD-LA followed by 2 min of 808 nm laser irradiation (1.226 W). These findings demonstrated that the PPy-RhB-PDA-CD-LA enabled effective control over drug release. Consequently, the undesired side effects on healthy tissues may be alleviated owing to reduced systemic DOX uptake, as a significant proportion of DOX can be released locally in the target region under dual stimulation by low pH and NIR light.

### 3.6. Cell Uptake and Intracellular Temperature Sensing

**Figure 12:**
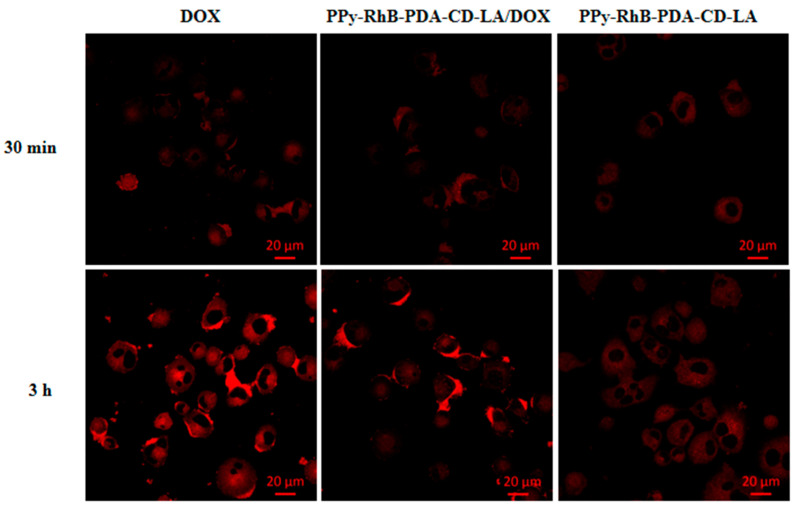
Cellular uptake of PPy-RhB-PDA-CD-LA/DOX, PPy-RhB-PDA-CD-LA, and free DOX after incubation for 30 min and 3 h. Scale bar: 20 µm.

A fluorescence microscope was used to observe and analyze the cell uptake behavior of PPy-RhB-PDA-CD-LA/DOX into SMMC-7721 cells after different incubation times in comparison with free DOX and PPy-RhB-PDA-CD-LA. Confocal images showed red fluorescence in the cytoplasm for the free DOX group at 30 min, and then the red fluorescence progressively accumulated into cell nuclei with prolonged incubation time to 3 h (Figure 12). In contrast, the fluorescence intensity of RhB in cells treated with PPy-RhB-PDA-CD-LA remained weak even at 3 h, indicating slower cellular internalization of the nanoplatform. In the PPy-RhB-PDA-CD-LA/DOX group, red fluorescence was initially observed in the cytoplasm. As time elapsed, the signal intensified and approached the nucleus, suggesting cellular uptake of PPy-RhB-PDA-CD-LA and subsequent sustained release of DOX. The cellular uptake mechanism was further validated through nuclear staining of SMMC-7721 cells using Hoechst 33258 (blue fluorescence). As shown in the merged images (Figure S7), PPy-RhB-PDA-CD-LA/DOX exhibited distinct colocalization of red fluorescence with blue nuclear staining, confirming effective internalization of PPy-RhB-PDA-CD-LA and triggered release of DOX into the nuclei. These results demonstrated that PPy-RhB-PDA-CD-LA facilitated efficient cellular uptake and controlled intracellular drug release.

**Figure 13:**
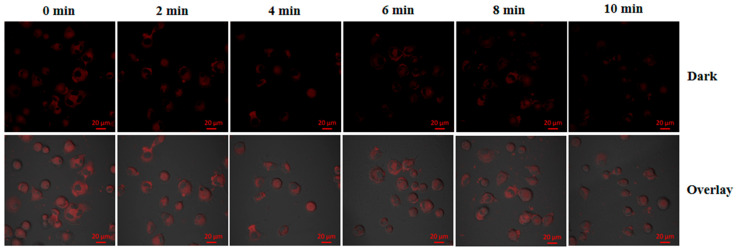
CLSM images of SMMC-7721 incubated with PPy-RhB-PDA-CD-LA after irradiation over different time periods. Scale bar: 20 µm.

To evaluate the performance of PPy-RhB-PDA-CD-LA within cells, heat production induced through NIR irradiation was chosen to promote the intracellular temperature. As shown in Figure 13, the red fluorescence intensity decreased significantly with increasing temperature in SMMC-7721 cells incubated with PPy-RhB-PDA-CD-LA. The photothermal effect generated by PPy-RhB-PDA-CD-LA induced gradual attenuation of RhB’s red fluorescence, which is consistent with temperature-dependent spectral variations. The fluorescence stability of PPy-RhB-PDA-CD-LA was systematically assessed under varying pH (3.0–9.0) and ionic strength. As shown in Figure S5, the fluorescence signal remained highly stable with negligible fluctuations under physiologically relevant conditions, confirming that intracellular microenvironmental changes do not significantly interfere with temperature sensing. In addition, the nanoplatform exhibited excellent photostability under continuous laser irradiation for one hour, with no appreciable signal decay (Supplementary Figure S5), indicating that photobleaching-induced signal loss is negligible throughout the experimental period. These results directly confirm the feasibility of using PPy-RhB-PDA-CD-LA for intracellular temperature monitoring. It should be noted that the cellular uptake analysis of PPy-RhB-PDA-CD-LA in this study was qualitative in nature. Furthermore, due to its excitation and emission wavelengths in the visible range, PPy-RhB-PDA-CD-LA was limited to self-monitoring of photothermal ablation in vitro and required avoiding interference from the inherent fluorescence of the loaded anticancer drugs. Therefore, the design and synthesis of temperature sensors excitable by NIR light should be pursued to enable more effective self-monitoring of temperature changes induced by photothermal conversion materials [45]. Additionally, more rigorous investigation of the targeting mechanism (including quantitative uptake studies using flow cytometry) and the long-term stability in physiological buffers will be essential in subsequent research for in vivo applications.

## 4. Conclusions

In this study, a multifunctional PPy-RhB-PDA-CD-LA nanoplatform through a facile assembly of RhB, PPy, PDA, β-CD, and LA was constructed. Each component was strategically incorporated to impart distinct functionalities to the nanoplatform; RhB provided thermal sensitivity, PPy acted as the photothermal conversion agent, the cavity of β-CD and the surface of PDA facilitated drug loading, and LA was introduced as a potential targeting moiety toward hepatocellular carcinoma cells. This nanoplatform exhibited exceptional photothermal conversion efficiency (η = 51.7%) and high loading capacity for DOX (326.6 mg/g). Furthermore, the photothermal property enabled it to modulate drug release through an NIR light trigger, reducing the toxic side effects on normal tissues caused by systemic drug delivery. Meanwhile, due to its good thermal sensitivity of 1.44% °C^−1^, the cellular temperature change under laser irradiation could be monitored through CLSM. Although challenges, such as precise release control, real-time tracking, and in vivo imaging, remain to be addressed for clinical translation, the innovative fusion of multiple functional components in a single system offers a promising strategy for the development of smart liver-specific nanomedicine.

## Figures and Tables

**Table 1:** Hydrodynamic size of PPy-RhB-PDA measured through DLS analysis.

m_PPy-RhB_:m_DA_	1:0	50:1	25:1	10:1	5:1
Size (nm)	129.6	133.2	131.7	133.4	131.9
PDI	0.096	0.109	0.101	0.123	0.087

## Data Availability

The original contributions presented in this study are included in the article/Supplementary Materials. Further inquiries can be directed to the corresponding authors.

## Supplementary Materials

The following supporting information can be downloaded at https://www.mdpi.com/article/10.3390/ma18235313/s1: synthesis and characterization of PEI-CD-LA, analysis of size distribution in TEM images, calculation of photothermal conversion efficiency, temperature-sensing performance of different nanothermometers, and CLSM images. Figure S1: Synthesis of the PEI-CD-LA; Figure S2: The FT-IR spectra of LA, PEI-CD and PEI-CD-LA; Figure S3: ^1^H NMR spectrum and chemical structure of PEI-CD; Figure S4: The histograms of size distribution of PPy-RhB (A), PPy-RhB-PDA (B) and PPy-RhB-PDA-CD-LA (C); Figure S5: Effect of (A) ionic strength, (B) pH and (C) time intervals of irradiation with UV light on the fluorescence intensity of PPy-RhB-PDA-CD-LA; Figure S6: Linear time data versus-Lnθ obtained from the cooling period; Figure S7: Cellular uptake of PPy-RhB-PDA-CD-LA, PPy-RhB-PDA-CD-LA/DOX and free DOX for 3 h. The nuclei were stained with Hoechst 33258. Scale bar: 20 µm; Table S1: The temperature sensing performance of different nanothermometers; Table S2: Fitting results of drug-release model of PPy-RhB-PDA-CD-LA/DOX; References [33,46,47,48,49,50,51,52,53,54,55,56,57,58] are cited in the Supplementary Materials.
