# Supplementary material for: β-Cyclodextrin-Grafted Polypyrrole–Rhodamine B Nanoplatforms for Drug Delivery and Image-Guided Photothermal Therapy In Vitro
Source: Materials (Basel). 2025 Nov 25;18(23):5313. doi: 10.3390/ma18235313 (PMC12693271; doi:10.3390/ma18235313)
Supplement: Supplementary file 1 [file materials-18-05313-s001.zip › materials-3960536-supplementary.pdf]

β-Cyclodextrin grafted polypyrrole-rhodamine B nanoplatforms for drug delivery and image-guided photothermal therapy in vitro

Shasha Hong[1], Yuan Jiao[2], Ruyu Li[3], Peng Lei[3], Chuan Dong[3], Shang Guo[1]* and Shaomin Shuang[3]*

[1]Shanxi Institute for Functional Food, Shanxi Agricultural University, Taiyuan 030031, Shanxi, P.R. China; sshong@sxau.edu.cn (S.S. Hong); gs0351@sohu.com (S. Guo)

[2]College of Environment and Ecology, Taiyuan University of Technology, Jinzhong, 030600, Shanxi, China; jiaoyuan@tyut.edu.cn (Y. Jiao)

[3]College of Chemistry and Chemical Engineering, Institute of Environmental Science, Shanxi University, Taiyuan 030006, PR China; 1603732324@qq.com (R.Y. Li); penglei@sxu.edu.cn (P. Lei); dc@sxu.edu.cn (C. Dong); smshuang@sxu.edu.cn (S.M. Shuang)

*Correspondence: gs0351@sohu.com (S. Guo); smshuang@sxu.edu.cn (S.M. Shuang)

Synthesis of PEI-CD-LA

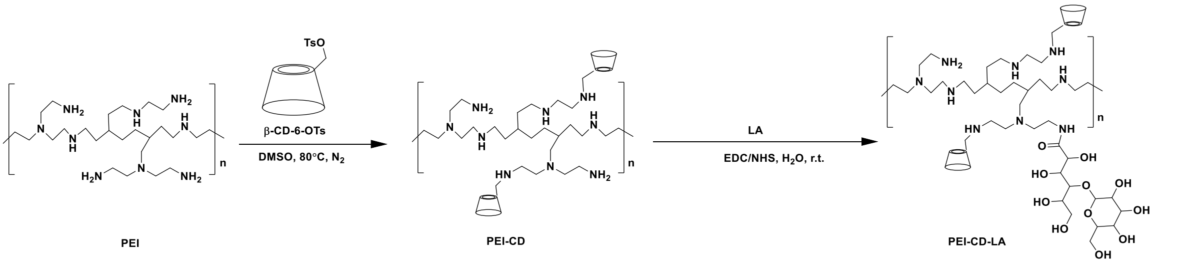

Fig. S1. Synthesis of the PEI-CD-LA.

PEI-CD-LA was synthesized with references to the reported procedure having some modifications[1, 2]. 4.36 g of 6-OTs-β-CD and 0.56 g of PEI (M.W. 1800) were dissolved into 100 mL of DMSO and then the mixture was refluxed at 80 °C for 5 days. Subsequently, the solution was dialyzed with the dialysis membrane of 1000 Da for 6 days and lyophilized to obtain the light-yellow powdery PEI-CD. Then PEI-CD was further functionalized with LA via the carbodiimide coupling reaction. LA (1 g, 2.8 mmol), NHS (0.77 g, 6.7 mmol), and EDC (1.28 g, 6.7 mmol) (LA/EDC/NHS molar ratio, 1:2.4:2.4) were dissolved in 50 mL of deionized water, then stirred for 4 h to activate the carboxylic groups of LA. After being added 0.2 g of PEI-CD, the mixture was stirred for another 24 h. The whole reaction process was carried out in the dark at room temperature. After the reaction completed, the solution was dialyzed with the dialysis membrane of 1000 Da for 2 days and lyophilized to obtain the white powdery PEI-CD-LA for subsequent experiments (Fig. S1).

Characterization of PEI-CD-LA

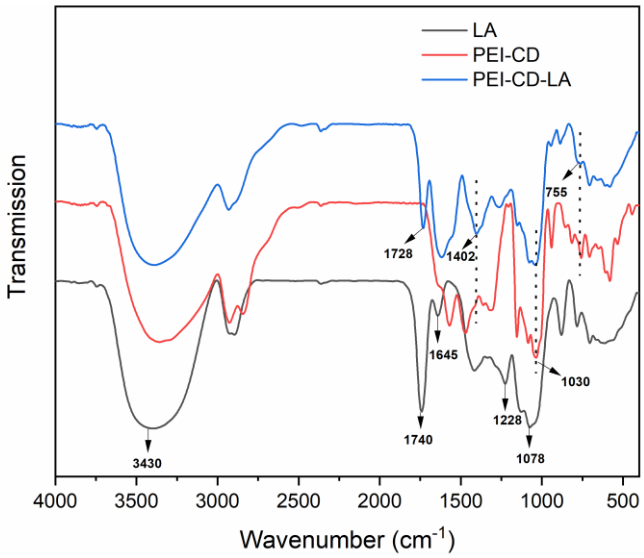

Fig. S2. (A) The FT-IR spectra of LA, PEI-CD and PEI-CD-LA.

FT-IR method was employed to confirm the formation of functional groups. The typical peaks of β-CD at 1030 cm^{-1} due to C-O stretching vibration, and the characteristic peaks of PEI at 1402 cm^{-1} and 755 cm^{-1} corresponding to vibration of -C-N and $-NH_2$, were also appeared in the FT-IR spectra (Fig. S2) of PEI-CD and PEI-CD-LA after the cross-linking reaction[3]. In addition, the typical -C=O stretching vibration (1740 cm^{-1}) of LA disappeared and new characteristic absorption peak (1728 cm^{-1}) of ester bond appeared after LA was modified on PEI-CD[4], signifying the successful conjunction of LA with PEI-CD.

^{1}HNMR Characterization of PEI-CD

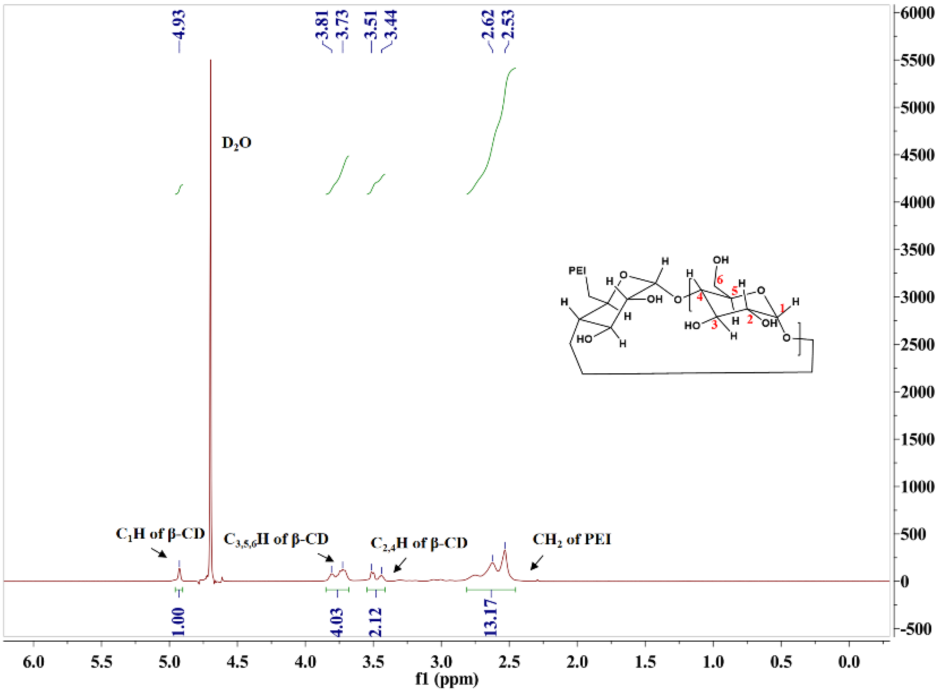

Fig. S3. ^{1}H NMR spectrum and chemical structure of PEI-CD.

As shown in Fig. S3, the ^{1}HNMR spectrum of PEI-CD consisted of peaks corresponding to the protons of PEI and β-CD, respectively. The degree of grafting of β-CD to PEI backone was calculated as follows:

$$\mathrm{CD-grafting\ level\ (\%)} = \frac{I_a/7}{I_b/4} * 100$$

Where I_a and I_b stand for the integration value of C_1H of β-CD and CH_2 of PEI, respectively. It was calculated that the degree of grafting of β-CD was about 4.3%. We could estimate that about 2 β-CD groups were conjugated onto every PEI chain based on the known degree of grafting of β-CD[5]. ^{1}H NMR (D_2O, 600 MHz): δH (ppm) = 4.93 (br, C_1H of β-CD), 3.73-3.81 (m, $C_{3,5,6}H$ of β-CD), 3.44-3.51 (m, $C_{2,4H}$ of β-CD), 2.53-2.62 (br, $-OCH_2-$ of PEI)。

Analysis of size distribution in TEM images

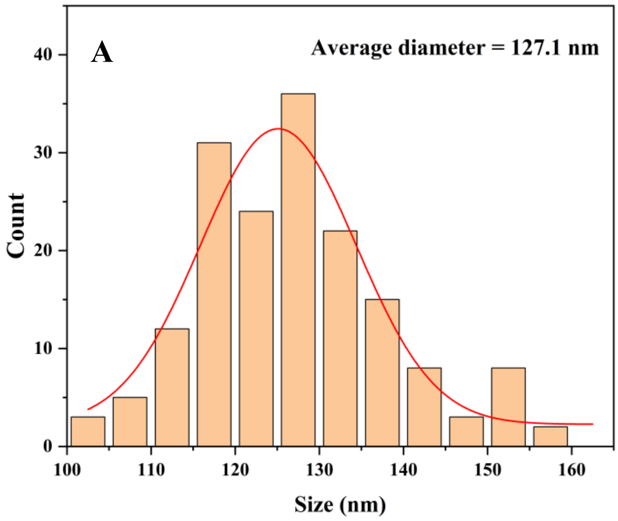

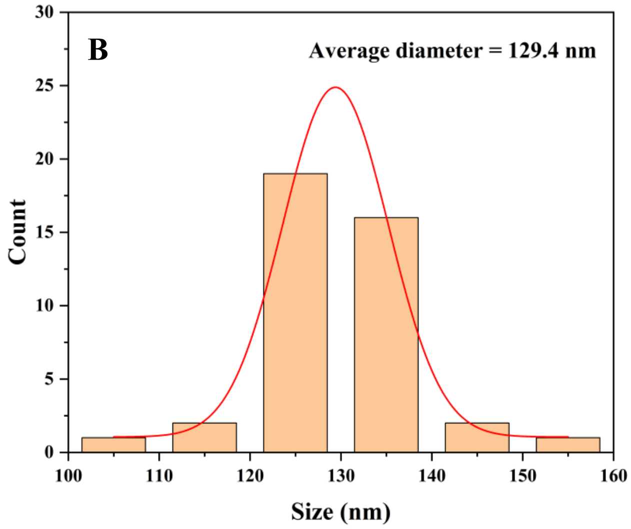

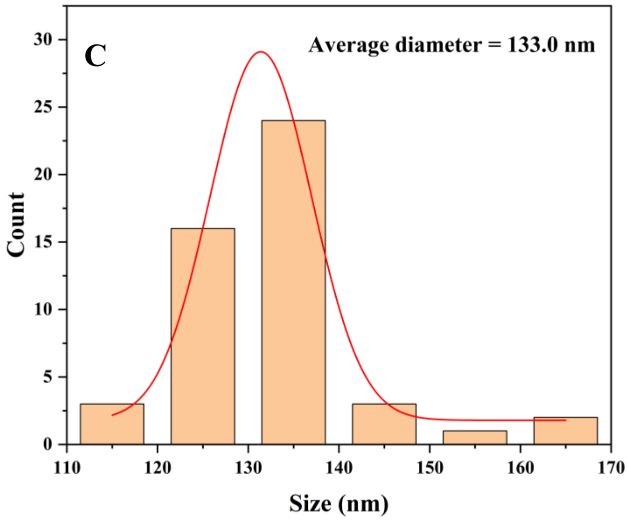

Fig. S4. The histograms of size distribution of PPy-RhB (A), PPy-RhB-PDA (B) and PPy-RhB-PDA-CD-LA (C).

The optical stability of PPy-RhB-PDA-CD-LA

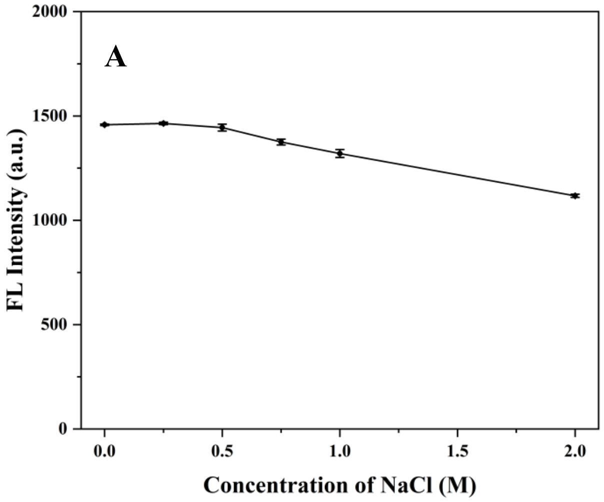

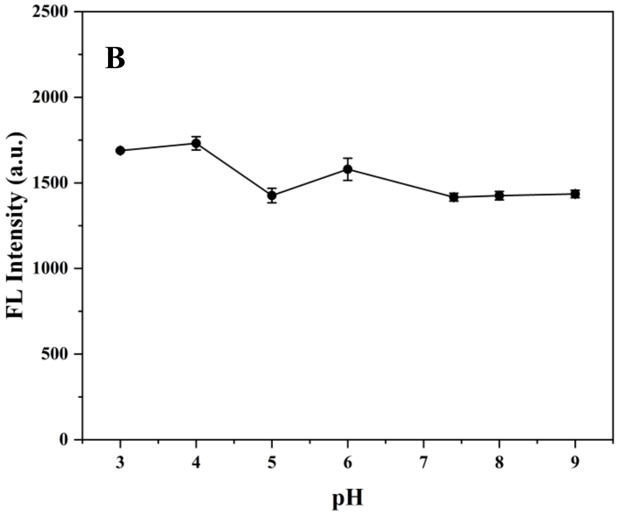

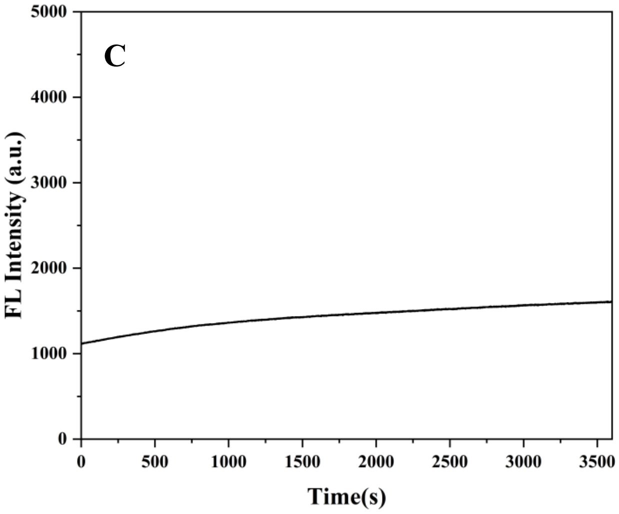

Fig. S5. Effect of (A) ionic strength, (B) pH and (C) time intervals of irradiation with UV light on the fluorescence intensity of PPy-RhB-PDA-CD-LA.

Temperature sensing performance

Table S1 The temperature sensing performance of different nanothermometers

Materials	Type	Detection range (°C)	S_R	Ref.
Yb^{3+}, Er^{3+}@DBCO	Lanthanide luminescent nanomaterials	25-43	1.4 % K^{-1}	[6]
TRF NPs	Polymer nanothermometers	25-65	2.37 % $°C^{-1}$	[7]
CDs	Carbon nanomaterials	23-45	7.9 % $°C^{-1}$	[8]
MOF-867@Eu^{3+}@CDs	Metal-organic frameworks	20-60	1.81 % K^{-1}	[9]
AuNCs@TPP	Metal clusters	21.3-39.2	2.8 % $°C^{-1}$	[10]
CPDs-AgNCs@PVA	Carbonized polymer dots-silver nanoclusters	20-80	/	[11]
emGFP-Mito	Green Fluorescent Protein	23-39	2.2 % $°C^{-1}$	[12]
PPy-RhB-PDA-CD-LA	Polymer nanoplatforms	20-60	1.44 % $°C^{-1}$	This work

Fitting results of drug-release model

Table S2 Fitting results of drug-release model of PPy-RhB-PDA-CD-LA/DOX

	Model	Equations	R^2
pH 7.4	Zero-order	Qt=0.3526t+19.0546	0.8330
	First-order	ln(100-Qt)=-0.0050t+4.3935	0.8634
	Higuchi	$Qt=3.4518t^{1/2}+13.4136$	0.9536
	Riter-Peppas	lnQt=0.2352lnt+2.7191	0.9931
pH 5.0	Zero-order	Qt=0.5215t+21.3568	0.9212
	First-order	ln(100-Qt)=-0.0084t+4.3713	0.9587
	Higuchi	$Qt=4.9302t^{1/2}+13.7327$	0.9832
	Riter-Peppas	lnQt=0.2629lnt+2.8464	0.9856
pH 5.0+NIR	Zero-order	Qt=0.5396t+31.3545	0.8947
	First-order	ln(100-Qt)=-0.0107t+4.2417	0.9451
	Higuchi	$Qt=5.0974t^{1/2}+23.4821$	0.9535
	Riter-Peppas	lnQt=0.2068lnt+3.2727	0.9642

Calculation of the photothermal conversion efficiency

The photothermal conversion efficacy (η) is calculated according to the previous method[13, 14], using the following equations:

$$\sum_i m_i C_{p,i} \frac{dT}{dt} = Q_{NPs} + Q_S - Q_{loss} \quad (1)$$

where m and C_p represent the mass and heat capacity of water, respectively, T represents the temperature of solution, Q_S is the heat from the light absorbed by the solvent and cuvette,

Q_{NPs} represents the photothermal energy input by PPy-RhB-PDA-CD-LA under 808 nm laser irradiation:

$$Q_{NPs} = I(1 - 10^{-A_{808}})\eta \quad (2)$$

where I is the laser power used in the photothermal experiment, η refers to the photothermal conversion efficiency, A^{808} is the absorbance of PPy-RhB-PDA-CD-LA at 808 nm.

Q_{loss} is the heat dissipation to the surroundings:

$$Q_{loss} = hS(T - T_{amb}) \quad (3)$$

$$Q_s = Q_{loss} = hS(T_{max,H_2O} - T_{amb}) \quad (4)$$

where h is the heat transfer coefficient, S is the surface area of the cuvette, T is the maximum steady-state temperature, T_{amb} is the ambient temperature of the environment.

At the maximum steady-state temperature, the thermal energy input is equal to the thermal energy lost.

$$Q_{NPs} + Q_S = Q_{loss} = hS(T_{max} - T_{amb}) \quad (5)$$

whereT_{max}represents the maximum of PPy-RhB-PDA-CD-LA under 808 nm laser irradiation.

According to the Eq.2 and Eq.4, η of PPy-RhB-PDA-CD-LA can be determined:

$$\eta = \frac{hS(T_{max} - T_{amb}) - hS(T_{max,H_2O} - T_{amb})}{I(1 - 10^{-A_{808}})} \quad (6)$$

In the above equation, only *hS* is unknown, which can be calculated by introducing θ:

$$\theta = \frac{T - T_{amb}}{T_{max} - T_{amb}} \quad (7)$$

The time constant (τ_s) for heat transfer from the system can be calculated referring to the following equation:

$$\tau_s = \frac{\sum_i m_i C_{p,i}}{hS} \quad (8)$$

Substituting Eq.7 into Eq.1 and giving the following expression:

$$\frac{d\theta}{dt} = \frac{1}{\tau_s}\left[\frac{Q_{NPs} + Q_S}{hS(T_{max} - T_{amb})} - \theta\right] \quad (9)$$

When the laser was shut off, the Q_{NPs}+Q_S=0，Eq.9 changed to：

$$dt = -\tau_s \frac{d\theta}{\theta} \quad (10)$$

$$t = -\tau_s \ln\theta \quad (11)$$

Thus, τ_s is calculated to be 293.40 by analyzing the linear time data (600s) from the cooling period vs -lnθ (Fig. S4). In the photothermal experiment, the mass of water is 1.5×10^{-3} kg, and the mass of PPy-RhB-PDA-CD-LA is 1.2×10^{-6} kg. Since the heat of water is higher than other materials, the m_{NPs} and C_{NPs} can be ignored. Substituting the mass (1.5×10^{-3} kg) and heat capacity (4.2×10^{3} J Kg^{-1} °C^{-1}) of

water into Eq.8, and *hS* was found to be 0.0215. The laser power is 1.226 W, and the absorbance at 808 nm was measured as 0.613. Utilizing this above equations and information, the η of PPy-RhB-PDA-CD-LA is calculated to be 51.7%.

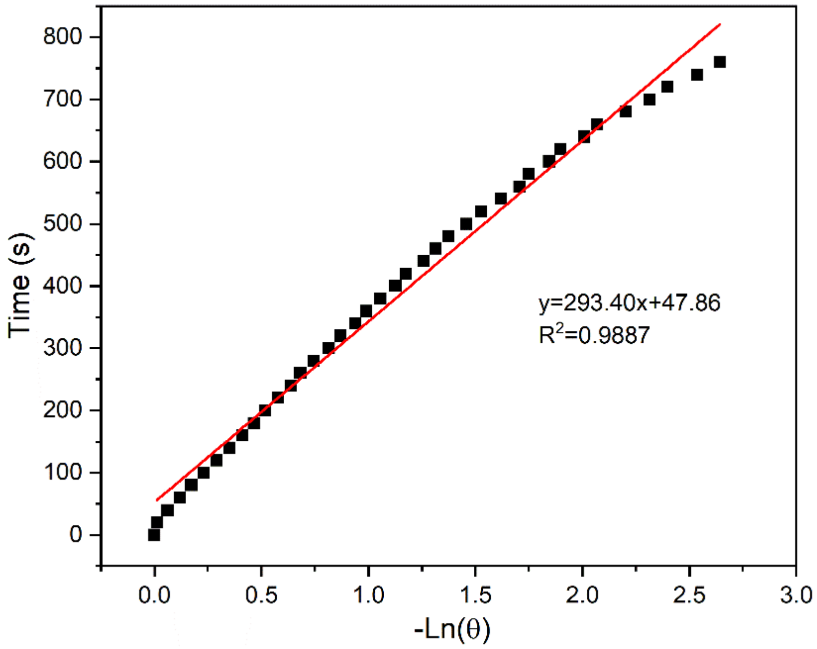

Fig. S6. Linear time data versus-Lnθ obtained from the cooling period.

Cell uptake

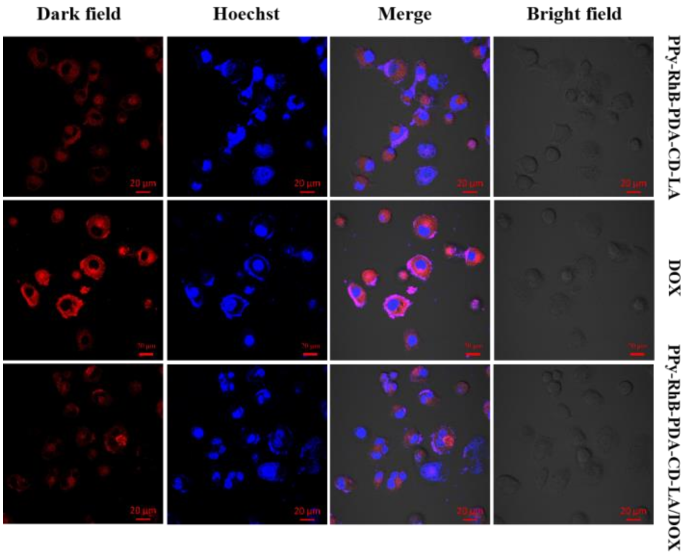

Fig. S7. Cellular uptake of PPy-RhB-PDA-CD-LA, PPy-RhB-PDA-CD-LA/DOX and free DOX for 3 h. The nuclei were stained with Hoechst 33258. Scale bar: 20 μm.

References

1. Pun, S. H.; Bellocq, N. C.; Liu, A.; Jensen, G.; Machemer, T.; Quijano, E.; Schluep, T.; Wen, S.; Engler, H.; Heidel, J.; Davis, M. E., Cyclodextrin-modified polyethylenimine polymers for gene delivery. *Bioconjugate chemistry* **2004,** 15, (4), 831-840.
2. Zhang, J.; Sun, H.; Ma, P. X., Host-guest interaction mediated polymeric assemblies: multifunctional nanoparticles for drug and gene delivery. *ACS nano* **2010,** 4, (2), 1049-59.
3. Liu, J.; Liu, G.; Liu, W.; Wang, Y.; Xu, M.; Wang, B., Turn-on fluorometric β-carotene assay based on competitive host-guest interaction between rhodamine 6G and β-carotene with a graphene oxide functionalized with a β-cyclodextrin-modified polyethyleneimine. *Microchimica Acta* **2016,** 183, (3), 1161-1168.
4. Hua, Z.; Zhang, X.; Chen, Y.; Liu, R.; Li, Y.; Li, J.; Liu, D.; Tan, M., A bifunctional hepatocyte-mitochondrion targeting nanosystem for effective astaxanthin delivery to the liver. *Food Chemistry* **2023,** 424, 136439.
5. Ping, Y.; Liu, C.; Zhang, Z.; Liu, K. L.; Chen, J.; Li, J., Chitosan-graft-(PEI-β-cyclodextrin) copolymers and their supramolecular PEGylation for DNA and siRNA delivery. *Biomaterials* **2011,** 32, (32), 8328-8341.
6. Liang, H.; Yang, K.; Yang, Y.; Hong, Z.; Li, S.; Chen, Q.; Li, J.; Song, X.; Yang, H., A Lanthanide Upconversion Nanothermometer for Precise Temperature Mapping on Immune Cell Membrane. *Nano Letters* **2022,** 22, (22), 9045-9053.
7. Meng, L.; Jiang, S.; Song, M.; Yan, F.; Zhang, W.; Xu, B.; Tian, W., TICT-Based Near-Infrared Ratiometric Organic Fluorescent Thermometer for Intracellular Temperature Sensing. *ACS Appl Mater Interfaces* **2020,** 12, (24), 26842-26851.

8. Silva, S. F. V.; Figueiredo, G.; Pereira, R. F. P.; de Zea Bermudez, V.; Fu, L.; André, P. S.; Carneiro Neto, A. N.; Ferreira, R. A. S., Time-gated multi-dimensional luminescence thermometry via carbon dots for precise temperature mobile sensing. *Nanoscale* **2024,** 16, (44), 20532-20541.
9. Li, Y.; Xiao, X.; Wei, Z.; Chen, Y., A Ratio Fluorescence Thermometer Based on Carbon Dots & Lanthanide Functionalized Metal-Organic Frameworks. *Zeitschrift für anorganische und allgemeine Chemie* **2022,** 648, (9), e202100323.
10. Wang, Y.; Liang, S.; Mei, M.; Zhao, Q.; She, G.; Shi, W.; Mu, L., Sensitive and Stable Thermometer Based on the Long Fluorescence Lifetime of Au Nanoclusters for Mitochondria. *Analytical Chemistry* **2021,** 93, (45), 15072-15079.
11. Sun, M.; Li, P.; Wang, M.; Liang, Y.; Yang, X.; Pang, S., Carbonized polymer dots-silver nanoclusters nanocomposite with dual-emission for property ratiometric fluorescence and visual detection of temperature. *Diamond and Related Materials* **2024,** 141, 110559.
12. Savchuk, O. A.; Silvestre, O. F.; Adão, R. M. R.; Nieder, J. B., GFP fluorescence peak fraction analysis based nanothermometer for the assessment of exothermal mitochondria activity in live cells. *Scientific Reports* **2019,** 9, (1), 7535.
13. Liu, Y.; Ai, K.; Liu, J.; Deng, M.; He, Y.; Lu, L., Dopamine-Melanin Colloidal Nanospheres: An Efficient Near-Infrared Photothermal Therapeutic Agent for In Vivo Cancer Therapy. *Advanced Materials* **2013,** 25, (9), 1353-1359.
14. Prakash, A.; Yadav, S.; Saxena, P. S.; Srivastava, A., Development of folate-conjugated polypyrrole nanoparticles incorporated with nitrogen-doped carbon quantum dots for targeted bioimaging and photothermal therapy. *Talanta* **2024,** 278, 126528.
